# Drivers of illicit alcohol consumption among at-risk populations aged 15–29 years old in Zambia: a qualitative perspective

**DOI:** 10.3389/fpubh.2025.1444334

**Published:** 2025-04-01

**Authors:** Tulani Francis L. Matenga, Cosmas Zyambo, Masauso Moses Phiri, Richard Zulu, Musawa Mukupa, Kumbulani Mabanti, Anna Hainze, Dhally M. Menda, Angela Rizzo, Ahmed Ogwell, Fastone M. Goma, Tom Achoki

**Affiliations:** ^1^Department of Health Promotion and Education, School of Public Health, University of Zambia, Lusaka, Zambia; ^2^Center for Primary Care Research, Lusaka, Zambia; ^3^Department of Community and Family Medicine, School of Public Health, University of Zambia, Lusaka, Zambia; ^4^Department of Pathology and Microbiology, School of Medicine, University of Zambia, Lusaka, Zambia; ^5^Independent Researcher, Nairobi, Kenya; ^6^School of Postgraduate Studies, University of Zambia, Lusaka, Zambia; ^7^Churches Health Association of Zambia (CHAZ), Lusaka, Zambia; ^8^AB InBev Foundation, New York, NY, United States; ^9^United Nations Foundation, New York, NY, United States; ^10^Africa Institute for Health Policy, Nairobi, Kenya

**Keywords:** illicit alcohol, young people, social-ecological model, drivers, Zambia

## Abstract

**Background:**

Illicit alcohol consumption is a major public health problem in Zambia and many other countries in Sub-Saharan Africa. This study aimed to determine drivers of illicit alcohol consumption among at-risk populations, such as youths, in selected urban areas in Zambia, using the social-ecological model as a lens to understand these drivers.

**Methods:**

Through a qualitative approach, we conducted semi-structured interviews with 12 key stakeholders from the Ministry of Health, Zambia Revenue Authority, local council public health departments, and rehabilitation centers. We also interviewed 30 alcohol consumers who frequent alcohol retail settings such as bars or liquor stores. Participants were purposively selected based on their knowledge of illicit alcohol consumption.

**Results:**

Social demographic factors such as age and sex were identified as key drivers. Adolescents as young as 10 years old initiated illicit consumption, with males constituting the majority of consumers. Limited job opportunities and recreational facilities in communities encouraged young people to consume illicit alcohol; this is worsened by the availability of alcohol in their social circles. Peer influence was also found to be a major driver of illicit alcohol consumption, as cultural norms normalized alcohol use. Lack of productive activities and economic disparities were also found to drive consumption among this group. Low-income individuals sought cheaper alternatives, such as home-distilled spirits or fermented alcohol, due to their affordability and availability in disadvantaged neighbourhoods. Social norms, such as the consumption of homemade alcoholic beverages during social gatherings such as weddings, were also significant drivers.

**Conclusion:**

Despite policies and state agencies banning illicit alcohol sale and production, the sale of alcoholic beverages to young people and weak enforcement of regulations across the country, especially in urban areas increases young people’s risk of consuming illicit alcohol. Applying the social-ecological model emphasizes the need for multi-level interventions. These interventions should target individuals, communities, and policy levels. Specifically, they should aim to regulate alcohol consumption, disrupt the social environments that promote illicit alcohol consumption, and ultimately facilitate positive behavior change among young people.

## Introduction

Globally, there are nearly three in 10 older adolescents (26.5%) aged 15–19 reported ever consuming alcohol (1). Alcohol use can begin as early as 15 years old, despite legal restrictions in many countries prohibiting the purchase, sale, and consumption of alcohol by individuals under the age of 18 (2). Countries with such restrictions include; Uganda (Liquor Act, 1960), Tanzania (Law of Child Act, 2009), Sudan (Child Act, 2010), South Africa (Children’s Act, 2005), and Seychelles (Children’s Act, 1982) (3). There is a growing trend in the production and consumption of illicit alcohol in many low- and middle-income countries (4). Illicit alcohol defined as alcoholic beverages (distilled or fermented) produced outside legal frameworks (5) is a significant concern in Africa. The WHO (6) reports that one-third of all alcohol consumed in the region is “unrecorded” amounting to 1.8 L per capita, per year. This often includes home-brewed beverages made from fermented or distilled grains, fruits, sugar cane, honey, or palm trees.

The burden of alcohol-related diseases is particularly high among low-income and middle-income country populations. This is exacerbated by high injury rates and limited implementation of public health policies and prevention strategies (5, 7). In 2019 alone alcohol consumption was responsible for 22,000 deaths and 1.1 million disability-adjusted life years (DALYs) in the Middle East and North Africa regions (8). It is estimated that the percentage of current drinkers below the age of 25 in Zambia was 44% in 2014 and 53% among those aged 15–19 years in 2018 (1). The Zambia Demographic Health Survey further indicated that the prevalence of alcohol consumption among adolescents is 32.0% of male adolescents aged 15–19 and 11.8% of female adolescents of the same age (9). Socioeconomic determinants, access to alcohol, lack of parental involvement, and peer pressure are some of the factors that contribute to the vulnerability of underage alcohol consumption among young people in Zambia (10). Illicit alcohol consumption among under aged individuals in the country has also led to high mental health issues, contributing to 80% of psychiatric illnesses (11), despite awareness of its negative effects (48).

A quantitative study conducted in Zambia showed that 80% (158 of the 196) of adolescents and young adults reported consuming alcohol (10). Other studies in the region have focused on associations between alcohol consumption and respondent sex, peer pressure, family history of alcohol abuse, unstable employment, poor social and coping skills, increased alcohol availability, and positive expectations regarding alcohol use (12, 13). Meanwhile, socio-demographic factors such as age, residence, and education level have also been studied as risk factors (14–16).

Few studies have used a comprehensive approach to determine drivers of illicit alcohol consumption among at-risk populations, such as youths in Zambia, as most studies have explored the factors associated with alcohol consumption in general (5, 10, 17). There is a need for a deeper understanding of the drivers of illicit alcohol consumption using a qualitative approach. This study therefore aimed to determine drivers of illicit alcohol consumption among at-risk populations aged 15–29 years old, in selected urban areas in Zambia, using the social-ecological model as a lens to understand these drivers.

### A social-ecological approach

To understand the drivers of illicit alcohol consumption among at-risk populations aged 15–29 years old in Zambia, this study utilized Bronfenbrenner's (18) Social-Ecological framework. This framework, widely recognized for its holistic approach to human behavior, emphasizes the interconnectedness between individuals and their environments. By examining both immediate surroundings and broader social contexts—including interpersonal relationships, community environments, and societal influences—the framework provides a comprehensive understanding of how these factors contribute to illicit alcohol consumption.

The individual/intrapersonal level consists of biological and predisposing factors such as age, sex, knowledge, behavioral intentions, and motivation while the interpersonal level includes interactions among those closest to the individual such as family, peers, relatives, and teachers (19). The physical environment consists of organizational and community-level determinants, including home, school, health centers, clubs, markets, and other settings while the societal level represents sociocultural determinants that shape the individual’s behaviors, including religion, ethnicity, socioeconomic status, marketing, public policy, and regulation (19). The Ecological Model has been considered an acceptable framework to link individual and social behaviors with environmental determinants, to help reduce serious and prevalent health problems by understanding multi-level factors that can influence people’s behavior (20). The importance of using the Social Ecological Model in this study is the consideration of interactions between people’s behavior and the environment (sociocultural, policy, and physical) (20). The Social Ecological Model states that behavior is affected by the interaction of multifaceted determinants such as intra-personal, inter-personal, and environmental influences hence the application of the social ecological model as a framework can help in understanding the drivers of illicit alcohol consumption.

## Methodology

### Study design

The study adopted a qualitative case study design using in-depth interviews to determine drivers of illicit alcohol consumption among at-risk populations aged 15–29 years old in Zambia. This was led by the investigators with the assistance of local trained research assistants.

### Study sites

The study was conducted in three urban areas in Zambia. The selection of the study sites was purposeful and included Lusaka, the capital city of Zambia, host to major trading activities in the country and location for major stakeholders such as policy makers. Ndola town a major urban center on the Copperbelt province was selected because it is also the industrial and commercial center of the Copperbelt province with major trading activities and also the third largest city in the country in terms of population size. Ndola town has in the past reported to have high consumption of illicit alcohol due to undeclared (tax leakage) production of alcohol and smuggling (21). Livingstone was also purposefully sampled as a border town for transit of illicit alcohol, the town shares its border with Namibia, Botswana, and Zimbabwe. Livingstone is the tourist hub of the country and also a trading town with various foreign trading activities. Livingstone has also in the past reported to have high consumption of illicit alcohol due to undeclared (tax leakage) production of alcohol and cross border smuggling.

### Study participants

In-depth interviews (IDIs) were conducted with purposefully selected consumers, and stakeholders, e.g., the Zambia Revenue Authority (ZRA), regulators such as the public health department at each city council, representatives from the psychiatric department at provincial hospitals and/or alcohol dependency rehabilitation centers, as well as other key stakeholders as necessary or cited from the literature and the mapping exercise.

### Sample size and recruitment of participants

Forty-two participants were interviewed; these included 12 key informant interviews with stakeholders and 30 in-depth interviews with alcohol consumers purposively selected from the three study sites. Letters were written to organizations requesting stakeholders who could provide more details on the drivers of illicit alcohol consumption among at-risk populations aged 15–29 years old. Organizations identified an individual knowledgeable about the topic and provided the researchers with the contact details for an interview to be arranged at their convenience. Alcohol consumers were purposefully sampled with the help of research assistants who were members of the communities where data was collected and were able to identify individuals who consume alcohol in their communities. A purposeful sample strategy was employed to attract potential participants through a maximum sampling approach that involved deliberately selecting a diverse range of participants to capture a wide range of perspectives on the drivers of illicit alcohol consumption among at-risk populations aged 15–29 years old. The sample was determined by theoretical saturation. A point when no additional themes or insights emerge from the data collection, and all conceptual categories have been explored, identified, and completed (22).

Policy makers were included in the study if they were knowledge about illicit alcohol consumption and also played a part in policy, regulatory or involved in the management of alcohol related health problems. Consumers were included in the study if they were involved in illicit alcohol consumption and had lived in the study site for at least 1 year. Participants that were unable to give consent to take part in the study due non comprehension of the informed consent were excluded.

### Data collection

Data was collected between October and November of 2023. Experienced interviewers conducted the interviews using semi-structured interview guides developed using the social-ecological model. The main outline of the interview guides included questions on socioeconomic and demographic factors contributing to illicit alcohol consumption, cultural and social norms that influence the acceptance and consumption of illicit alcohol in certain communities or regions among certain populations, public health concerns regarding the consumption of spirits that are illegally manufactured, the drivers of illicit alcohol consumption including availability, affordability and accessibility of illicit alcohol in the community and regulation of illicit alcohol production. The interviews were conducted at a private location of the participant’s choosing to ensure confidentiality, which included places of work, home locations, or a central location and lasted between 20 and 60 min. A voice recorder was used to ensure that the data collected was accurate and credible. Participants were each assigned a unique identification number that was used to capture data, this ensured that their identity was protected. A letter of introduction was written to introduce the researchers and the topic of discussion to the different individuals, prior arrangements were then made for interviews with participants.

### Data analysis

Once all the data was transcribed and checked for accuracy, it was then uploaded into Nvivo version 12, a qualitative software program, for qualitative data management and analysis (23). The study used a thematic analysis approach to analyse the data and to guide the analysis process, an initial deductive coding scheme was developed using the social-ecological model as a framework. This coding scheme was then discussed and agreed upon by the team. Once the coding scheme was drafted, codes and their definitions were uploaded into the software to facilitate the first layer of coding and to identify additional deductive codes from the data. Analysis steps, according to Creswell (24) were followed.

The Nvivo 12 bundle project, with all the transcripts and codes, was then created. Inductive codes were used to capture high-level interpretation and emergent themes. An extensive list of codes, which included the first deductive codes from the framework and inductive codes and their definitions, was then developed after the establishment and application of an initial code list. These final lists of codes were then applied to all the transcripts in the software. Using Nvivo 12, query reports (codes linked to quotations from transcripts were summed up in major themes) and created, representing recurrent themes related to the objectives of the study.

## Results

To determine drivers of illicit alcohol consumption among at-risk populations, such as youths, in selected urban areas in Zambia, in-depth interviews were conducted with various stakeholders and bar patrons. A total of 42 interviews were conducted across the three geographies. In total, 12 stakeholders and 30 alcohol consumers were interviewed (see Table 1).

**Table 1:** List of participants in the study.

Type of participants	Approach	Number of interviews per city	Total number of participants
Stakeholders	KIIs	4	12
Patrons (consumers)	IDIs	10	30
Total interviews			42

The social-ecological model was adapted as a holistic and multi-layered approach to understanding the complex issues driving underage alcohol consumption. Table 2 below provides a summary of themes that were developed from the interviews, and which guided the coding process of the transcripts.

**Table 2:** Factors associated with illicit alcohol consumption among youths.

Major themes	Subthemes
Individual level factors	Age at alcohol initiation
	Sex
Interpersonal level factors	Peer influence
	Social norms
	Lack of productive activities
Societal level factors	Economic disparities
	Cultural norms
	Family influence
Environment level factors	Affordability
	Accessibility
	Availability
Policy level factors	Inadequate enforcement
	Loopholes

### Individual level factors

Interviewees from both the local authorities and health personnel revealed that several demographic factors were associated with the consumption of illicit alcohol among at-risk populations aged 15–29 years old. Demographic factors that served as drivers to illicit alcohol consumption include;

*Age at alcohol initiation*: according to insights from multiple stakeholders, a significant portion of illicit alcohol consumption occurs among young people residing in medium- to high-density urban areas. This underscores the crucial role of age in driving the consumption of illicit alcohol among young people who are vulnerable and not in a position to make important decisions.

*In my line of duty, I have noticed that most of this alcohol is sold to the young, the youth, largely between the ages of 15–30, …, and most of these are male. So even when you ask the manufacturer (of the alcohol), “Where do you supply this alcohol?.” They will tell you that*. [KII with Policymaker 1].

*The other factor I think is the age at which many young people begin to abuse illicit alcohol during their teenage hood, when they are still emotionally vulnerable, and not mature enough to make those critical decisions about themselves. So, age group is a factor*. [KII with Ministry of Health Official 1].

Patrons also revealed that they began consuming alcohol at a very young age which has continued into their adult years. Thus, initiating alcohol consumption at a young age has significantly contributed to their enduring struggle with addiction into adulthood.

*I started quite young when I was 17 years old…… Sometimes I drink throughout the week, it depends on how I am feeling, this whole week, I have been drinking, and even today I am drinking.* [30–34-year-old Female Patron].

*Sex*: Most of the interviewees revealed that illicit alcohol consumption is more commonly associated with males, although they mentioned that this gap has been narrowing in recent years with an increase in females also consuming illicit alcohol.

*I have noticed that most of these consuming illicit alcohol in our communities are mostly males. [KII with Policymaker].*

*I think boys or males are more likely to consume alcohol because they have more freedom than the girl child*. [KII with Ministry of Health official].

Officials from local councils indicated that even though males are the largest consumers of illicit alcohol most producers of illicit alcohol are females.

*And on the part of brewing, you will find that the people doing the brewing are females.* [KII with Health Inspector].

### Intrapersonal level factors

Intrapersonal-level factors play a significant role in driving at-risk populations to engage in illicit alcohol consumption. These factors encompass various characteristics and traits that predispose individuals to substance use and shape their decision-making processes.

*Peer influence:* The influence of peers was seen as an important driver of illicit alcohol consumption. These influences were described in different ways including actively encouraging or pressuring others to participate in illicit alcohol consumption and or direct persuasion or coercion.

*Yes, but then peer pressure, where you try to stop, but then a friend asks you to sip, from sipping to just drinking*. [35–39-year-old Male Patron].

*Social norms*: illicit alcohol consumption is often normalized within social groups, participants revealed that some of these norms exist within their own families and those closest to them;

*There are certain social norms that are very much okay with indulging in these things and the whole family, like even in the villages, when we go, I do not think my grandfather or grandmother will stop me from drinking* illicit alcohol [KII with ministry of health official].

*Lack of productive activities*: Joblessness was reported as a driver of illicit alcohol consumption. Patrons revealed that during idle time, young people seek activities to pass the time. Some turn to illicit alcohol consumption as a form of entertainment or distraction.

*I think it is because most people have no jobs, no resource centers to keep them busy, or Trade Centers to help them learn a trade and start something like their own business and make them entrepreneur*s. [25–29-year-old Male Patron].

*People go to drink because they do not have anything to do*. [KII with Health Inspector 1].

*There is nothing to do, there is nowhere to go and if I go to a friend’s place, he will give me some alcohol and will drink and that’s because I have nothing to do. If for instance, you were my friend, and you came to pick me up so we do something, I would not find time to drink, but if I had nothing to do, we would both be drinking.* [25–29 -year-old Male Patron].

### Societal level factors

Societal-level factors are said to exist at the level of society and influence the consumption of illicit alcohol, which operates through a variety of mechanisms. Interviewees, including those from the health sector and regulatory bodies, cited these societal-level factors as follows:

*Economic disparities*: communities with significant income disparities experience higher rates of illicit alcohol consumption, as individuals with limited resources are inclined to seek out cheaper alternatives. Some participants noted that home-based alcohol production and consumption characteristically occurred in areas experiencing poverty. Participants also revealed that individuals from high-density, low-income communities are more prone to consume illicit alcohol.

*It is often those who come from low-income settings that we see consuming illicit alcoholic beverages such as home brews. The places are generally characterized by poor housing and this is where you will find the low class living close together*. [KII with Policymaker 1].

Additionally, economic hardship can lead to limited access to employment opportunities, making youth more susceptible to illicit alcohol consumption. Several participants revealed that low income levels are one of the reasons why many adolescents and youths turn to illicit alcohol.

*The most hit are the lowly paid, they obviously gravitate toward cheaper sources, like Kachasu, boxer, you know, those which are produced in smaller sachets, or bottles, they are cheaper like I said, these drinks are cheaper than soft drinks.* [KII with Ministry of Health official 2].

*Cultural norms*: cultural events and gender roles fostered specific norms around illicit alcohol. These norms are further entrenched by long-standing traditions and, in some communities, by the economic importance of alcohol production and sales.

*Alcohol is acceptable in Zambian situations and Zambian cultures, and in rural areas, young people start drinking at a very young age, 13 or 14. I have been to different rural areas, doing research and other work in the Southern province, and I found a tavern where they only sell Gankata, which is a local brew. In the Northern province, the same brew is sold, and similarly in small taverns, specifically for 7 days. In the Eastern province, it’s the same; 7 days is sold.* [KII with Ministry of Health official 2].

*Family influence*: furthermore, the consumption of alcohol by family members such as parents normalizes the consumption among young people.

*Coming from homes where parents or relatives consume alcohol. It’s also a risk factor for the offspring to consume*. [KII with Ministry of Health official 3].

### Environmental level factors

Interviewees from the health sector described the drivers of illicit alcohol consumption from an environmental level; these factors affect an individual’s access to illicit alcohol and their ability to afford it.

*Availability*: illicit alcohol is readily available in the community, sometimes even sold openly where people live and on the streets. This availability of illicit alcohol has made it more convenient for young consumers.

*Illicit alcohol, currently, … is everywhere, everyone is selling it, and it is all over*. [KII with Rehab Centre Manager].

*Yes, very easy, because this is the alcohol found in makeshift stores, and then Kachasu is brewed in homes*. [30–34-year-old Female Patron].

Additionally, illicit alcohol is normally found in high-density areas where taverns and shebeens (informal on-site alcohol retailers) are a common practice. Taverns and shebeens are often the only places where people in high-density areas socialize and are often willing to sell illicit alcohol to young people because it is cheaper and more profitable than legitimate alcohol.

*Illicit alcohol is mainly brewed in high-density areas, at low cost because people can afford to rent, they can afford to set up their low-cost illicit brewing business there and the people around that area tend to know those areas*. [KII with Health official].

*Affordability*: illicit alcohol is often available at cheaper prices compared to legally produced and taxed alcohol in many communities. This affordability makes it an appealing option for individuals, especially young people who may not have the financial resources to buy legally produced alcoholic drinks which may be more expensive.

*And then the reason could be you know in the rural places or communities around, where we live, many people are not aware of the effects and dangers. It is locally brewed where they live because it’s cheap and brewed where they live*. [KII with Rehab Centre Manager].

*The other driver is the price, illicit alcohol is very cheap and very affordable. It’s cheaper than soft drinks*. [KII with Health official].

The rise in illicit alcohol production activities in the community was also mentioned by participants as being a result of the need for income generation for the family. The price of alcohol, was described as cheap and an alternative to legally produced beverages.

*And then the reason could be you know in the rural places or communities around, we live in, many people are not aware of the effects and dangers of locally brewed alcohol where they live because it’s cheap and brewed where they live.* [KII with Rehab center Manager].

*Yes, very easy, because this is the alcohol found in makeshift stores, and then Kachasu is brewed in homes*. [30–34-year-old Female Patron].

*Accessibility*: accessibility to illicit alcohol was widely discussed as a driver for consumption. Proximity to premises producing and selling illicit alcohol was seen as a common feature of many neighbourhoods.

*It is that easy and accessible, so people access it a lot and it is cheap and you can even get it for free*. [KII with rehab centre].

### Policy level factors

At the policy level, interviewees from authorities including the Zambia Revenue Authority and local government discussed several policy-level factors driving the consumption of illicit alcohol among young people. The Liquor Licensing Act of 2011 forbids the sale or delivery of intoxicating liquor to a child (a person under 18 years of age). This policy further aims to balance promoting responsible drinking with protecting public health from alcohol-related harm.

*Inadequate enforcement*: despite the availability of the above Act, lack of government regulation and oversight, and weak or ineffective regulatory systems regarding alcohol production and distribution, were described as major drivers of illicit alcohol consumption by participants. This challenge results from weak regulatory systems and limited manpower for reinforcement, which may lower the likelihood of holding individuals or entities accountable for illicit alcohol production and distribution.

*It is because of our lack of proper enforcement. I would say, enforcement is there, but adequate, let me use the word inadequate enforcement*. [KII with regulator 1].

*Fortunately, the laws in Zambia are very good but we do not have enforcers, I think some of the best laws that control alcohol in Africa, Zambia has them, if you read CAP 167, very good law if I am selling alcohol and you are already drunk and you are in my bar, I am not supposed to sell you alcohol* [KII with Ministry of Health official].

These weak regulations are worsened by logistical challenges faced by local authorities in monitoring illicit alcohol production in the communities. Interviewees from the authorities reported that they often have limited resources to devote to monitoring illicit alcohol trade and consumption due to limited manpower, funding, and equipment.

*The challenges are many, number one is financial challenges in terms of logistics where we can say monitoring instruments such as vehicles to go and monitor and inspect it is a challenge for the local authorities*. [KII with regulator 2].

*Loopholes*: loopholes exist for small-scale or home-based alcohol production, which can be exploited for illicit purposes. In Zambia, small-scale producers take advantage of the lack of designated areas for manufacturing and therefore produce alcohol in their homes without the authorities’ knowledge.

*Some countries have designated places where to manufacture alcohol from and there are certain routes that they use to produce alcohol. So, if you do not produce alcohol in those designated premises, then it is easy for the authorities to catch you, but from our end, anyone can produce alcohol from anywhere, so those are loopholes, for me that is the loophole. We need to control the production aspect and the supply aspect*. [KII with Policy maker 1].

## Discussion

Our study, which employed the social-ecological framework to explore drivers of illicit alcohol consumption among at-risk youth aged 15–29 years old in selected Zambian towns, revealed several key factors influencing this behavior across multiple levels of influence. These key drivers included social demographic factors like age and gender, lack of employment opportunities, and peer influence. Alcohol consumption is part of young people’s social norms, with group culture normalizing alcohol use and economic disparities driving consumption. Low-income individuals often seek cheaper alternatives, such as home-produced or illegally distilled spirits, due to affordability and availability in disadvantaged neighbourhoods (25).

### Individual level factors

Individual level factors including age are a driver of illicit alcohol consumption. Being young is often linked to being immature, this immaturity can make young people more susceptible to risky decision-making (26). In Zambia, the initiation of young people into illicit alcohol has led to dependency and addiction giving rise to young people who are dependent on alcohol and feared by the community. Being male also drives young people to consume illicit alcohol. Kyei-Gyamfi et al. (27) found that males are more likely to consume alcohol than females, attributing this to the cultural expectations of masculinity leading to the high prevalence of alcohol consumption among males. However, our study found that this gap has been narrowing in recent years with an increase in females also consuming illicit alcohol as revealed by other studies (28). This also suggests that the epidemiology of alcohol use is changing in young people (29).

Our finding on sex and age as drivers of illicit alcohol consumption is similar to that of Zyaambo et al. (30) who revealed that the factors considered to be associated with alcohol consumption were age and sex. Social expectations and cultural norms often differ regarding alcohol consumption for males and females. In many cultures, alcohol consumption is more socially acceptable for males, while females may face greater social stigma or disapproval (27). These norms can influence drinking patterns between males and females. Additionally, our study reveals that there is a rise in females involved in illicit alcohol production.

### Interpersonal level factors

Our results highlight that peer influence is a major driver of illicit alcohol consumption. Young people are heavily influenced by values and beliefs through relationships with others and thus are influenced by their peers to consume illicit alcohol, which is a contributing factor to alcohol and drug abuse (31, 32). This result supports other studies that found peer influence to be a major cause of alcohol consumption among young people (33, 34). One explanation for the influence of others is that young people often seek a sense of belonging that promotes identity, self-definition, and shared values, standards, and behaviors, thereby improving their understanding of themselves in connection to others (35).

We also found that alcohol consumption was part of young people’s social norms. When some members of a group consume alcohol, other members may be influenced to initiate alcohol consumption due to group culture. MacArthur et al. (36) demonstrated how the wider alcohol culture normalized alcohol use for young people, helping to generate perceptions of drinking as a social practice associated with being “cool,” mature, and popular as well as an important means of enhancing enjoyment and social status. Family influence is another factor that drives illicit alcohol consumption, when family members normalize alcohol use in the family. Social norms are an important driver of illicit alcohol consumption among young people. Most African traditions involve the consumption of homemade alcohol during social gatherings within the community, embedding the practices of alcohol production and sales as an important part of society.

### Community level factors

Economic disparities are another driver of the consumption of illicit alcohol. Our study found that individuals from low-income communities with limited resources are inclined to seek out cheaper alternatives such as illicit distilled or fermented alcohol. Other studies reveal that lower-income individuals are major consumers of illicit alcohol, likely due to their affordability and availability in disadvantaged neighbourhoods (37). Participants in a study by Skylstad et al. (38) argue that while the intake of substances was generally high in the study setting, it was higher in poor neighborhoods and slums, where brewing spots were common. Easy access to illicit alcohol due to widespread availability in communities, including home-brewed spirits encouraged young people to consume illicit alcohol. Additionally, illicit alcohol is often more affordable than legally produced alcohol, making it more accessible to low-income individuals as others have observed (39).

### Societal level factors

Our study further reveals that the high concentration of liquor stores increases the physical availability of alcohol, and the heavy promotion of alcohol makes heavy drinking more socially acceptable and encourages consumption in disadvantaged neighbourhoods, similar to what was found by Hull et al. (40). Meanwhile, Mtumbi et al. (41) found that middle-income individuals exhibited lower odds of harmful alcohol use, indicating potential disparities across socioeconomic strata. This aligns with our findings showing how economic status influences illicit alcohol consumption. Studies also show that most adolescents drink illicit distilled spirits, especially those produced in homes because they are cheaper and easier to obtain than illicit spirits sold at stores (42).

Cultural groups and entire societies have created various norms for defining the appropriate use of alcohol (43). Our findings support those of others, who have similarly highlighted that alcohol use is a regular part of society, with alcohol consumption common at social settings, parties, sporting events, and celebrations both inside and outside the home (44, 45). Similarly, we found that drinking was a part of social events, and drinking alone was seen as unusual and a cause for concern. Others have noted the lack of employment opportunities which leads to feelings of emptiness and meaninglessness thus leading to illicit alcohol consumption (46).

### Policy level factors

Our results also revealed that policies and state agencies regulating illicit alcohol sales and production are available and in existence. Despite these measures (legislation policies, and state agencies), the sale of both illicit and licit alcoholic beverages to young people such as those aged 15–29 years old is a common phenomenon. Also, there is weak enforcement of regulations on selling alcoholic beverages to young people in both urban and rural areas, making them more susceptible to consuming alcohol, our study found the existence of gaps in the regulatory system. Policies aimed at regulating and controlling the production and sale of illicit alcohol are essential to reduce its availability and affordability. Even though the Zambia Act of 2011 and the National Alcohol Policy of 2018 forbid the sale and distribution of alcohol to people younger than 18, our study reveals a lack of adherence to alcohol regulatory measures within communities, particularly regarding opening and closing hours and easy accessibility. This finding is similar to that of Mungandi et al. (10). Furthermore, Crane et al. (47) argue that though Zambia possesses a robust legal discourse surrounding alcohol, the lack of enforcement of these policies—such as the age of drinking, the prohibition on unlicensed facilities, or the hours at which bars may open—contributes to the availability of these spaces for consumption. This calls for further strengthening and strict adherence to the policy on alcohol and sensitization especially to traders of alcohol.

## Limitations

This study has some limitations, being qualitative in nature with a small sample size, findings from this study may not be generalizable to other communities that may not be similar to the context in which we conducted the study. Further, the study is prone to social desirability as participants could have given responses that they felt the interviewer wanted to hear. Despite these limitations, our findings resonate with other studies conducted in other countries. Future research involving a larger, more geographically diverse sample could provide a more comprehensive picture.

## Conclusion

Illicit alcohol consumption poses a significant public health challenge in Zambia, particularly among vulnerable populations. This qualitative study provides valuable insights into the social, economic, and cultural drivers behind this behavior. The findings highlight several key factors influencing illicit alcohol consumption among at-risk youth; individual-level factors such as age and sex emerged as significant drivers. Young males particularly those initiating alcohol consumption early in life, were found to be more susceptible. At the interpersonal level, notably peer pressure, significantly impacted alcohol consumption choices among young people. Social norms surrounding alcohol use, including within family settings, also played a crucial role. Societal-level factors such as unemployment and economic disparities were identified as significant contributors. Limited economic opportunities and the search for affordable distractions fuelled alcohol consumption, particularly among low-income populations. Environmental-level factors highlighted the accessibility and affordability of illicit alcohol. The widespread availability of illicit alcohol in communities, often at lower prices than legally produced beverages significantly increased consumption.

Policy level factors revealed significant weaknesses in the regulatory framework. Inadequate enforcement of existing laws and regulations, coupled with loopholes in the system, facilitated the growth of illicit alcohol consumption in Zambia. A multifaceted approach that addresses the root causes, promotes access to affordable formal alcohol and provides culturally appropriate harm reduction and treatment services are therefore essential to curb illicit alcohol consumption and improve public health outcomes in Zambia.

## Data Availability

The raw data supporting the conclusions of this article will be made available by the authors, without undue reservation.
