# Correction: Drivers of illicit alcohol consumption among at-risk populations aged 15–29 years old in Zambia: a qualitative perspective

**DOI:** 10.3389/fpubh.2025.1672215

**Published:** 2025-08-21

**Authors:** Tulani Francis L. Matenga, Cosmas Zyambo, Masauso Moses Phiri, Richard Zulu, Musawa Mukupa, Kumbulani Mabanti, Anna Hainze, Dhally M. Menda, Angela Rizzo, Ahmed Ogwell, Fastone M. Goma, Tom Achoki

**Affiliations:** ^1^Department of Health Promotion and Education, School of Public Health, University of Zambia, Lusaka, Zambia; ^2^Center for Primary Care Research, Lusaka, Zambia; ^3^Department of Community and Family Medicine, School of Public Health, University of Zambia, Lusaka, Zambia; ^4^Department of Pathology and Microbiology, School of Medicine, University of Zambia, Lusaka, Zambia; ^5^Independent Researcher, Nairobi, Kenya; ^6^School of Postgraduate Studies, University of Zambia, Lusaka, Zambia; ^7^Churches Health Association of Zambia (CHAZ), Lusaka, Zambia; ^8^AB InBev Foundation, New York, NY, United States; ^9^United Nations Foundation, New York, NY, United States; ^10^Africa Institute for Health Policy, Nairobi, Kenya

**Keywords:** illicit alcohol, young people, social-ecological model, drivers, Zambia

In the published article, there was a mistake in the affiliation of authors.

Tulani Francis L. Matenga^1,2^

Cosmas Zyambo^1,2^

Masauso Moses Phiri^1,2^

Richard Zulu^2^

Musawa Mukupa^2^

Kumbulani Mabanti^2^

Anna Hainze^3^

Dhally M. Menda^4,5^

Angela Rizzo^3,6^

Ahmed Ogwell^7^

Fastone M. Goma^2^

Tom Achoki^3,8^

**Institutional affiliations**

- ^1^Department of Health Promotion and Education, School of Public Health, University of Zambia, Lusaka, Zambia
- ^2^Center for Primary Care Research, Lusaka, Zambia
- ^3^Department of Community and Family Medicine, School of Public Health, University of Zambia, Lusaka, Zambia
- ^4^Department of Pathology and Microbiology, School of Medicine, University of Zambia, Lusaka, Zambia
- ^5^Independent Researcher, Nairobi, Kenya
- ^6^School of Postgraduate Studies, University of Zambia, Lusaka, Zambia
- ^7^Churches Health Association of Zambia (CHAZ), Lusaka, Zambia
- ^8^AB InBev Foundation, New York, NY, United States
- ^9^United Nations Foundation, New York, NY, United States
- ^10^Africa Institute for Health Policy, Nairobi, Kenya

The correct affiliations are as follows:

Tulani Francis L. Matenga^1,2^

Cosmas Zyambo^2,3^

Masauso Moses Phiri^2,4^

Richard Zulu^2^

Musawa Mukupa^2^

Kumbulani Mabanti^2^

Anna Hainze^5^

Dhally M. Menda^6,7^

Angela Rizzo^8^

Ahmed Ogwell^9^

Fastone M. Goma^2^

Tom Achoki^8,10^

**Institutional affiliations**

- ^1^Department of Health Promotion and Education, School of Public Health, University of Zambia, Lusaka, Zambia
- ^2^Center for Primary Care Research, Lusaka, Zambia
- ^3^Department of Community and Family Medicine, School of Public Health, University of Zambia, Lusaka, Zambia
- ^4^Department of Pathology and Microbiology, School of Medicine, University of Zambia, Lusaka, Zambia
- ^5^Independent Researcher, Nairobi, Kenya
- ^6^School of Postgraduate Studies, University of Zambia, Lusaka, Zambia
- ^7^Churches Health Association of Zambia (CHAZ), Lusaka, Zambia
- ^8^AB InBev Foundation, New York, NY, United States
- ^9^United Nations Foundation, New York, NY, United States
- ^10^Africa Institute for Health Policy, Nairobi, Kenya

The conflict of interest statement was erroneously given as “The authors declare that the research was conducted in the absence of any commercial or financial relationships that could be construed as a potential conflict of interest.”

The correct conflict of interest statement is “AR and TA declare that they are full-time employees of the AB InBev Foundation. AH was an independent consultant affiliated with AB InBev Foundation. The remaining authors declare that the research was conducted in the absence of any commercial or financial relationships that could be construed as a potential conflict of interest.”

The original version of this article has been updated.

